# The clinical utility of dynamic ctDNA monitoring in inoperable localized NSCLC patients

**DOI:** 10.1186/s12943-022-01590-0

**Published:** 2022-05-19

**Authors:** Yin Yang, Tao Zhang, Jingbo Wang, Jianyang Wang, Yang Xu, Xiaotian Zhao, Qiuxiang Ou, Yang Shao, Xin Wang, Yuqi Wu, Linfang Wu, Xin Xu, Kunpeng Xu, Jingjing Zhao, Luhua Wang, Nan Bi

**Affiliations:** 1 Department of Radiation Oncology, National Cancer Center/National Clinical Research Center for Cancer/Cancer Hospital, Chinese Academy of Medical Sciences and Peking Union Medical College, No.17 PanjiayuanNanli, Chaoyang District, Beijing, 100021 China; 2 Geneseeq Research Institute, Nanjing Geneseeq Technology Inc, Nanjing, Jiangsu China; 3 School of Public Health, Nanjing Medical University, Nanjing, 211166 China; 4 Department of Radiation Oncology, National Cancer Center/National Clinical Research Center for Cancer/Cancer Hospital & Shenzhen Hospital, Chinese Academy of Medical Sciences and Peking Union Medical College, 113 Baohe Road, Longgang District, Shenzhen, 518116 Guangdong Province China

## Main text

Approximately one-third of the non-small cell lung cancer (NSCLC) patients are diagnosed with inoperable localized disease [1], and definitive chemoradiotherapy (CRT) is the standard of care for these patients. Although the results of the PACIFIC trial, a randomized multi-centre phase III trial evaluating the role of anti-PD-L1 (programmed cell death-ligand 1) antibody in patients with unresectable stage III NSCLC, demonstrated significant and sustained survival benefits of durvalumab consolidation treatment after CRT [2], which has been used as the new standard of care in this setting. It is of clinical importance to find an effective biomarker to direct precise consolidation treatment after CRT.

Circulating tumor DNA (ctDNA) is a promising noninvasive prognostic biomarker to monitor disease progression, and it has been studied in multiple cancers [3–5]. For NSCLC, Chaudhuri et al. showed that patients with negative posttreatment ctDNA had significantly better clinical outcomes than those with positive posttreatment ctDNA [3]. As an increasing number of studies have tried to investigate the clinical utility of serial ctDNA in lung cancer, researchers have realized that the posttreatment ctDNA levels exhibit a dynamic pattern. Breadner et al. demonstrated that there was an acute burst of ctDNA release in NSCLC patients who received CRT, and the ctDNA level peaked approximately 4 hours after treatment [6]. Therefore, selecting appropriate ctDNA time point (TP) would facilitate the prediction of disease status and patient prognosis.

Here, we performed targeted next-generation sequencing (NGS) of 474 cancer-related genes to characterize serial plasma samples in NSCLC patients who received front-line CRT/radiotherapy (RT). To select the most informative time to collect liquid biopsy, we analyzed the ctDNA level of various time points and correlated them with patients’ clinical outcomes.

## Results and discussion

### Study design and patient characteristics

**Fig. 1:**
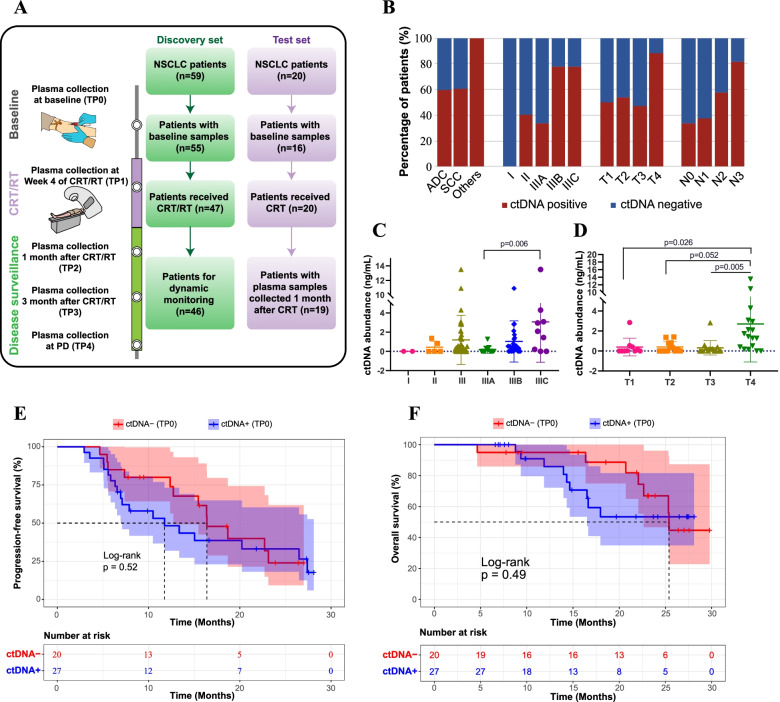
Baseline ctDNA status correlated with disease stage but not patient prognosis. **A** The workflow of the study. Specifically, TP0 plasma samples were collected before any treatments, TP1 plasma samples were collected in the middle of CRT/RT treatment (i.e., in the fourth week during CRT/RT), and other plasma samples were collected after CRT/RT treatment (i.e., TP2 for 1 month after CRT/RT, TP3 for 3 months after CRT/RT, and TP4 for the progressive disease after CRT/RT). **B** The relationship between baseline ctDNA status and TNM stage in NSCLC patients (*n* = 55). **C**-**D** The relationship between ctDNA abundance and disease stage (**C**) or T stage (**D**) in NSCLC patients (n = 55). Data are presented as the median +/− 2*IQR (interquartile range). The Bonferroni method was used for multiple comparison correction. **E**-**F** Kaplan-Meier curve of progression-free survival (**E**) or overall survival (**F**) stratified by baseline ctDNA status for patients in the discovery set

We prospectively enrolled 59 NSCLC patients in the discovery set to evaluate the clinical utility of serial ctDNA monitoring. After excluding 4 patients without pretreatment plasma samples, ctDNA data of the remaining 55 patients were included in the baseline analysis (Fig. 1A). Among these 55 patients, 45.5 and 50.9% were diagnosed with adenocarcinoma (ADC) and squamous cell carcinoma (SCC), respectively, and the majority of the patients (48/55) had locally advanced diseases (Supplementary Table 1). After collecting the baseline plasma samples from the 55 patients (TP0), 47 of them received CRT/RT. The prescribed CRT regimen was a platinum-based doublet recommended by the NCCN guidelines [7], including cisplatin plus etoposide, carboplatin plus paclitaxel, or cisplatin plus pemetrexed (non-squamous only). Despite the loss to follow-up and patient death, a considerable fraction of patients had their plasma samples collected during/post the CRT/RT treatment, including 39 patients in the fourth week during CRT/RT (TP1), 35 patients after 1 month of CRT/RT (TP2), 28 patients after 3 months of CRT/RT (TP3), and 13 patients at disease progression (PD) (TP4) (Fig. 1A). In addition, 20 external NSCLC patients who received CRT were included in the test set to validate the results from the discovery set, and the two datasets had comparable demographic and clinical features (Fig. 1A and Supplementary Table 1).

### Disease/tumor status, but not patient prognosis, was associated with baseline ctDNA

By sequencing the baseline plasma samples from the 55 patients, 34 (61.8%) had detectable ctDNA (Supplementary Table 2 and Supplementary Fig. 1). Intriguingly, we found that patents’ TNM stages were closely associated with baseline ctDNA detection (Fig. 1B and Supplementary Table 2). Particularly, patients with positive baseline ctDNA tended to have more advanced diseases (Fig. 1B). In addition to ctDNA detection status, we calculated the level of ctDNA according to previous studies [8]. The cell-free DNA (cfDNA) level was generally constant in patients with different TNM stages, whereas the ctDNA level showed an overall increasing trend when TNM stages increased (Fig. 1C, D and Supplementary Table 3). Therefore, the baseline ctDNA detection and ctDNA levels were positively related to disease stage, tumor size, and regional metastasis.

We then stratified patients according to their baseline ctDNA status. Although patients with negative baseline ctDNA tended to have better progression-free survival (PFS) and overall survival (OS) than those with positive baseline ctDNA, neither PFS nor OS reached statistical significance (Fig. 1E and F), implying that baseline ctDNA seemed not to be a good prognostic biomarker for CRT/RT-treated localized NSCLC patients.

### ctDNA collected 1 month after CRT/RT showed a superior capacity to predict patient prognosis

Next, we investigated the prognostic value of serial ctDNA that was collected during the treatment and/or during disease surveillance. During the course of the monitoring, the cfDNA level remained stable (Supplementary Fig. 2A). In contrast, the percentage of ctDNA-positive patients continued to decrease (Supplementary Fig. 2B), and the postreatment ctDNA levels were significantly reduced compared with the baseline, with the TP2 (i.e., 1 month after RT/CRT) having the lowest median ctDNA concentration (Supplementary Fig. 2C,D).

**Fig. 2:**
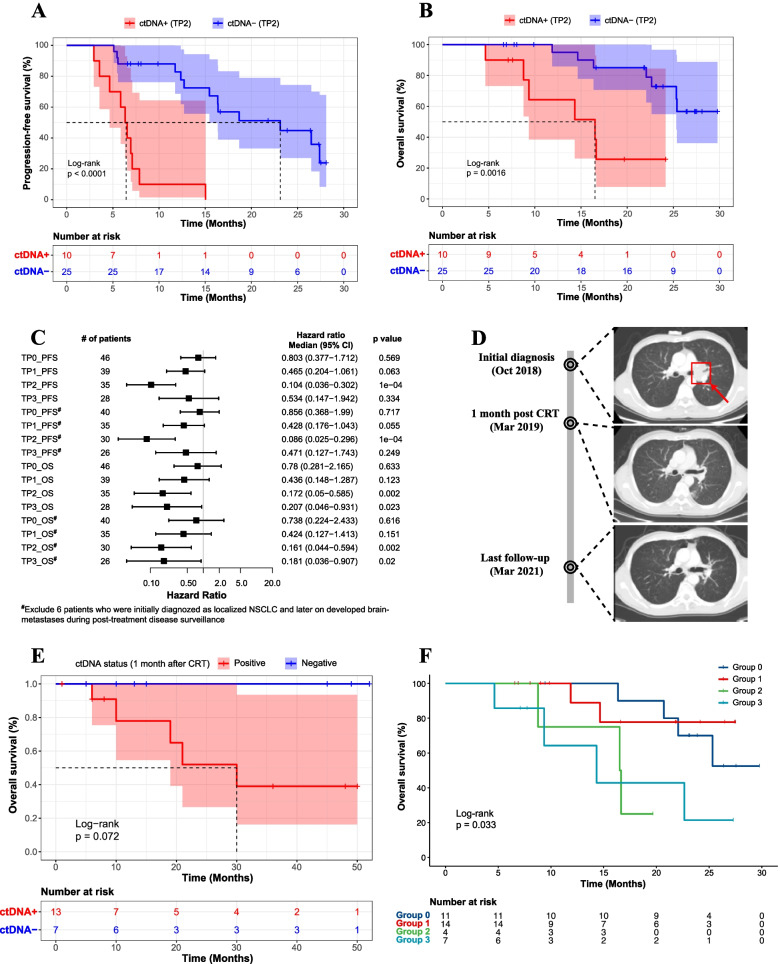
One month after CRT/RT was the optimal ctDNA time point to predict patient response. **A**-**B** Kaplan-Meier curve of progression-free survival (**A**) or overall survival (**B**) stratified by ctDNA detection status at TP2 in the discovery set. **C** Forest plot of the hazard ratio of various plasma sampling time points based on either progression-free survival (PFS) or overall survival (OS) in the discovery set. Patients with or without brain metastases were analyzed separately. Data are presented as median the +/− 95% CI (confidence interval). **D** A representative case of a male patient who had stage IIIA SCC and received definitive concurrent CRT. The red arrow indicates the initially diagnosed tumor, which became undetectable after CRT. **E** Kaplan-Meier curve of overall survival stratified by ctDNA detection status at 1 month post-CRT in the external test cohort. A total of 20 independent stage III NSCLC patients whose plasma samples were collected at baseline, in the fourth week during CRT, and after 1 month of CRT were included in the analysis. **F** Kaplan-Meier curve of overall survival stratified by ctDNA dynamic changes during disease surveillance in the discovery cohort

We then stratified patients based on ctDNA detection at time points TP1-TP3. Notably, these ctDNA time points demonstrated promising capacity for estimating patient prognosis compared with the baseline ctDNA (Fig. 2A,B and Supplementary Fig. 3). Strikingly, ctDNA positivity at TP2 showed a superior capacity to separate good and poor prognostic patients using all available patients in the discovery set (Fig. 2A,B). Because 87.2% of these patients had stage III disease and received CRT, we further verified our conclusion using exclusively stage III patients to minimize potential biases (*P* < 0.001 for the log-rank test; Supplementary Fig. 4A,B). Considering that some patients developed brain-metastases during postreatment disease surveillance and that the blood-brain barrier could potentially impair the release of the ctDNA into the peripheral blood, we analyzed the hazard ratio at various time points in patients with/without brain-metastases. Consistently, ctDNA detection at TP2 was still the optimal to estimate clinical outcomes post-CRT/RT regardless of the later-on brain metastasis status (Fig. 2C). Our observation was further demonstrated using a representative case of a male patient who had stage IIIA SCC and received definitive concurrent CRT (Fig. 2D). Specifically, the patient had a considerable level of baseline ctDNA at initial diagnosis, which became undetectable at TP2; correspondingly, his tumor shrank significantly after CRT and he remained progression-free for at least 28 months based on the last follow-up (Fig. 2D).

Our results were validated using the test set of 20 stage III NSCLC patients whose plasma samples were collected at baseline, in the fourth week during CRT, and after 1 month of CRT (Fig. 1A). Consistent with the results in the discovery set, patients with negative ctDNA detection at 1 month post-CRT tended to have a better PFS and OS than those with positive ctDNA detection (Fig. 2E and Supplementary Fig. 4C). Overall, these results suggested that 1 month after CRT/RT was the optimal TP to collect plasma samples for predicting patient prognoses.

### The prognostic value of the dynamic change in ctDNA levels

Based on ctDNA dynamic changes, we stratified the patients into 4 groups (Supplementary Fig. 5A-C): 1) patients with undetectable ctDNA in all tested samples (Group 0); 2) patients whose ctDNA was detectable at baseline and became undetectable before PD (Group 1); 3) patients who had decreased but detectable levels of ctDNA at the last follow-up before PD (Group 2); and 4) patients who had increased levels of ctDNA at the last follow-up before PD (Group 3). Notably, patients in Group 0 and Group 1 had better PFS and OS than those in Group 2 and 3 (Fig. 2F and Supplementary Fig. 5D). Additionally, the clearance of ctDNA at the last follow-up before PD was strongly associated with patients’ clinical outcomes (Supplementary Fig. 6). These results implied the potential prognostic value of ctDNA dynamic changes in inoperable localized NSCLC.

## Conclusions

In summary, we found that baseline ctNDA was highly correlated with disease stages, but it was a poor prognostic marker. In contrast, ctDNA collected 1 month post-CRT/RT was the optimal to predict patients’ PFS and OS, and the dynamic change in ctDNA was closely associated with clinical outcomes. Our results further expanded the clinical utility of ctDNA for the early estimation of prognosis and timely adjustment of treatment regimens in patients with inoperable localized NSCLC.

## Methods

Methods and materials used in our study are attached as Supplementary information.

## Supplementary Information

**Additional file 1: Supplementary Figure 1**. The baseline ctDNA genetic profile of adenocarcinoma (ADC) and squamous carcinoma (SCC) patients in the discovery set.

**Additional file 2: Supplementary Figure 2**. ctDNA level was at the lowest level after 1 month of CRT/RT treatment. A The cfDNA concentration of various plasma sampling time points in the discovery. B Bar plot of the positive ctDNA detection rate across various plasma collection times in the discovery. C The ctDNA abundance across various ctDNA time points in patients in the discovery cohort who had available plasma samples at the specific time point. Data are presented as the median +/− 2*IQR (interquartile range). The Bonferroni method was used for multiple comparison correction. D The ctDNA across various ctDNA time points in patients in the discovery cohort who had detectable ctDNA at the specific time point. Data are presented as the median +/− 2*IQR. The number of patients for each is listed below the figure. The Bonferroni method was used for multiple comparison correction.

**Additional file 3: Supplementary Figure 3**. The prognostic capacity of various ctDNA time points in the discovery set. A-B Kaplan-Meier curve of progression-free survival (A) or overall survival (B) stratified by ctDNA detection status at TP1. C-D Kaplan-Meier curve of progression-free survival (C) or overall survival (D) stratified by ctDNA detection status at TP3.

**Additional file 4: Supplementary Figure 4**. The prognostic value of the TP2 time point. A-B Kaplan-Meier curve of progression-free survival (A) or overall survival (B) stratified by ctDNA detection status at TP2 in stage III patients of the discovery set. C Kaplan-Meier curve of progression-free survival stratified by ctDNA detection status at 1 month post-CRT in the external test cohort. A total of 20 independent stage III NSCLC patients whose plasma samples were collected at baseline, in the fourth week during CRT, and after 1 month of CRT were included in the analysis.

**Additional file 5: Supplementary Figure 5**. Patient grouping was based on the dynamic change in ctDNA in the discovery cohort. A Group 1 of patients whose ctDNA was detectable at TP0/TP1 and was cleared at the last follow-up. B Group 2 of patients whose ctDNA was decreased but still detectable at the last follow-up. C Group 3 of patients whose ctDNA was increased at the last follow-up compared with baseline. D Kaplan-Meier curve of progression-free survival stratified by ctDNA dynamic changes during disease surveillance in the discovery cohort.

**Additional file 6: Supplementary Figure 6**. The clearance of ctDNA at the last follow-up was associated with patients’ clinical outcomes. A-B Kaplan-Meier curve of progression-free survival (A) or overall survival (B) stratified by ctDNA detection status at the last follow-up before disease progression in the discovery set.

**Additional file 7: Supplementary methods. Supplementary Table 1**. Demographic and clinical characteristics of the 55 NSCLC patients in the discovery set and 20 NSCLC patients in the test set. **Supplementary Table 2**. The relationship between baseline ctDNA detection and various clinical characteristics of the 55 NSCLC patients in the discovery set. **Supplementary Table 3**. The correlation between baseline ctDNA detection and clinical characteristics in 55 NSCLC patients of the discovery set.

## Data Availability

The datasets used and analyzed during the current study are available from the corresponding authors on reasonable request.

## Abbreviations

ADC: Adenocarcinoma
cfDNA: Cell-free DNA
CI: Confidence interval
CRT: Chemoradiotherapy
ctDNA: Circulating tumor DNA
IQR: Interquartile range
LA-NSCLC: Locally advanced NSCLC
NGS: Next-generation sequencing
NSCLC: Non-small cell lung cancer
OS: Overall survival
PD: Progressive disease
PD-L1: Programmed cell death-ligand 1
PFS: Progression-free survival
RT: Radiotherapy
SCC: Squamous cell carcinoma
TP: Time point
