# Supplementary figures and images for: The clinical utility of dynamic ctDNA monitoring in inoperable localized NSCLC patients
Source: Mol Cancer. 2022 May 19;21:117. doi: 10.1186/s12943-022-01590-0 (PMC9118575; doi:10.1186/s12943-022-01590-0)

# Supplementary Figure 1

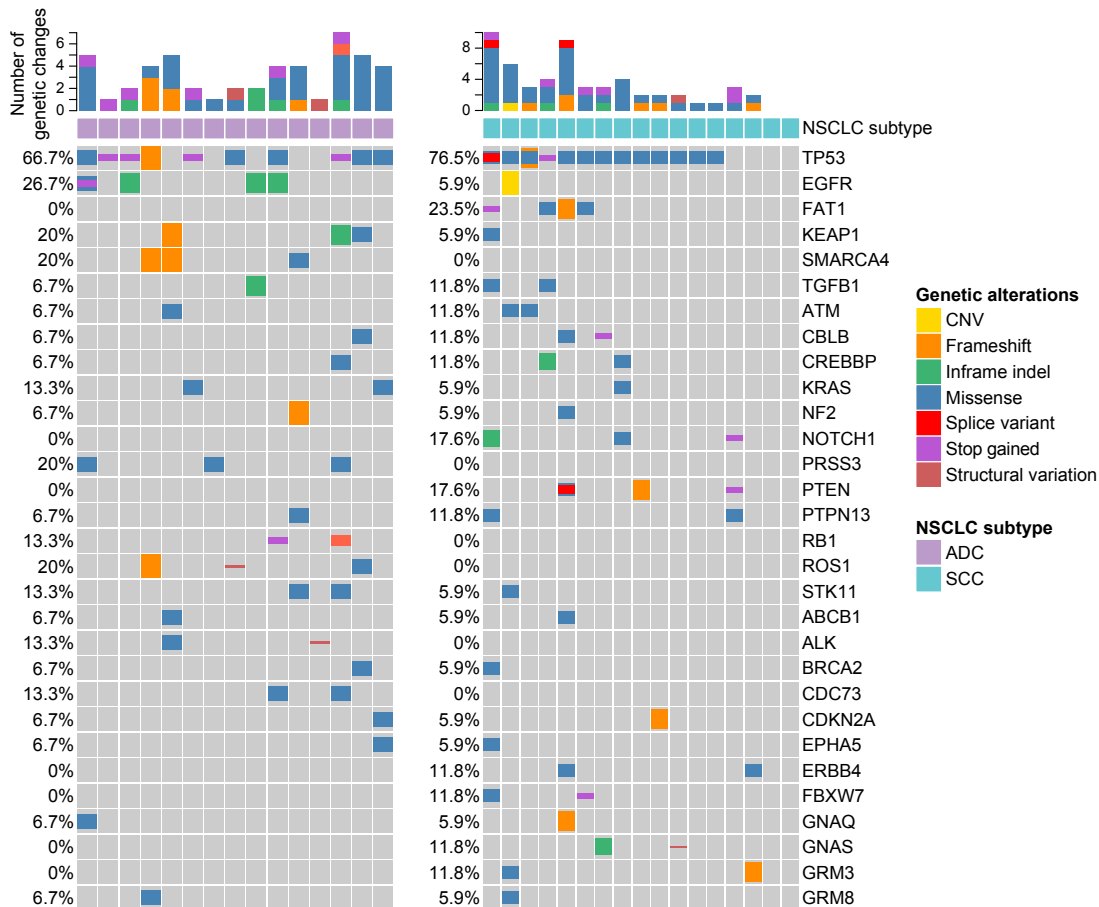

Supplement: Supplementary file 1 — Additional file 1: Supplementary Figure 1. The baseline ctDNA genetic profile of adenocarcinoma (ADC) and squamous carcinoma (SCC) patients in the discovery set. [file 12943_2022_1590_MOESM1_ESM.pdf]

## Supplementary Figure 2

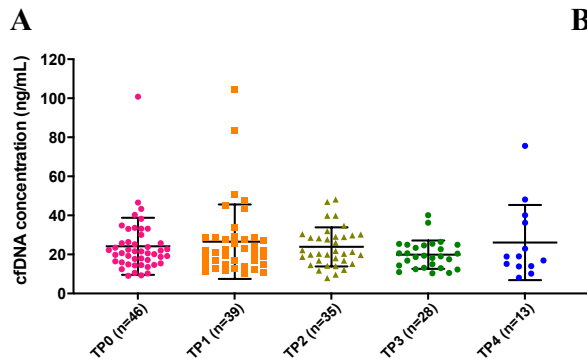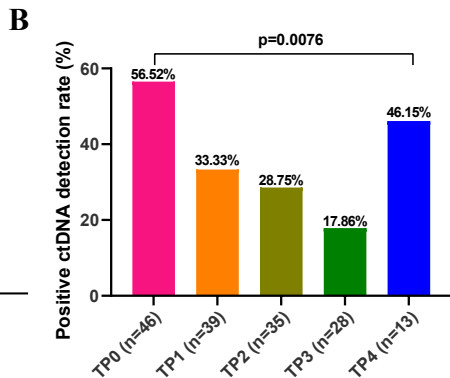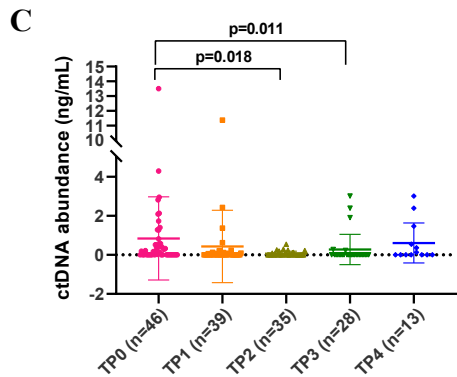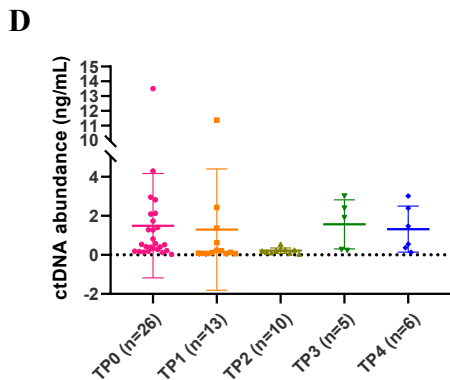

Supplement: Supplementary file 2 — Additional file 2: Supplementary Figure 2. ctDNA level was at the lowest level after 1 month of CRT/RT treatment. A The cfDNA concentration of various plasma sampling time points in the discovery. B Bar plot of the positive ctDNA detection rate across various plasma collection times in the discovery. C The ctDNA abundance across various ctDNA time points in patients in the discovery cohort who had available plasma samples at the specific time point. Data are presented as the median +/− 2*IQR (interquartile range). The Bonferroni method was used for multiple comparison correction. D The ctDNA across various ctDNA time points in patients in the discovery cohort who had detectable ctDNA at the specific time point. Data are presented as the median +/− 2*IQR. The number of patients for each is listed below the figure. The Bonferroni method was used for multiple comparison correction. [file 12943_2022_1590_MOESM2_ESM.pdf]

# Supplementary Figure 3

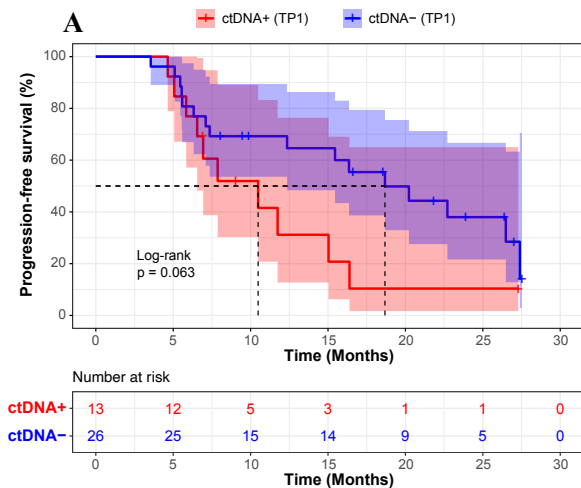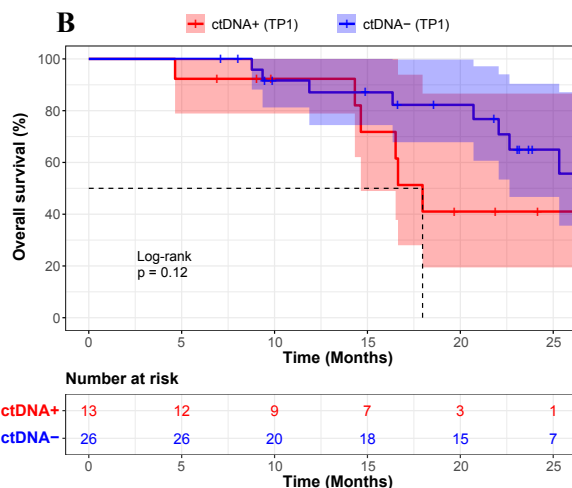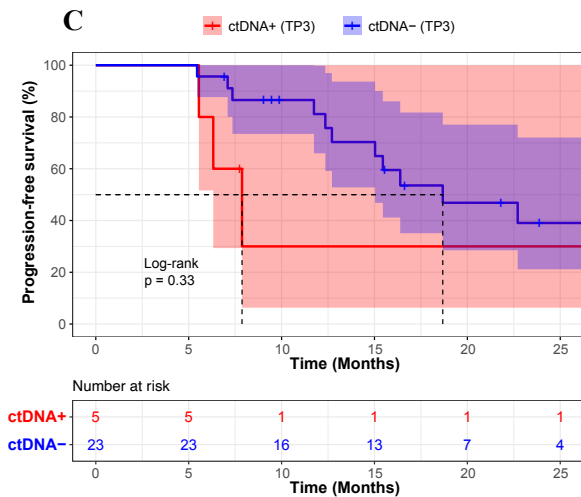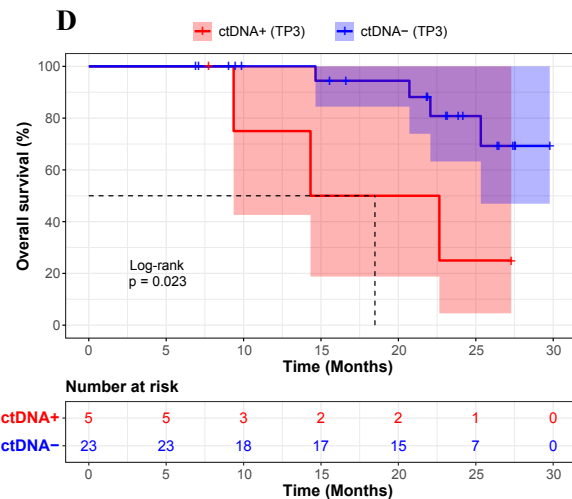

Supplement: Supplementary file 3 — Additional file 3: Supplementary Figure 3. The prognostic capacity of various ctDNA time points in the discovery set. A-B Kaplan-Meier curve of progression-free survival (A) or overall survival (B) stratified by ctDNA detection status at TP1. C-D Kaplan-Meier curve of progression-free survival (C) or overall survival (D) stratified by ctDNA detection status at TP3. [file 12943_2022_1590_MOESM3_ESM.pdf]

# Supplementary Figure 4

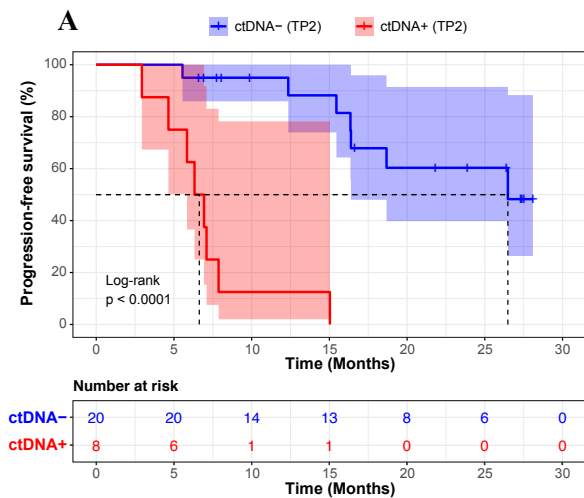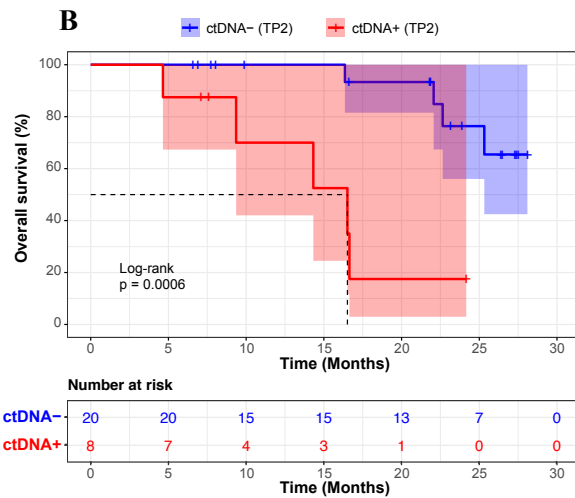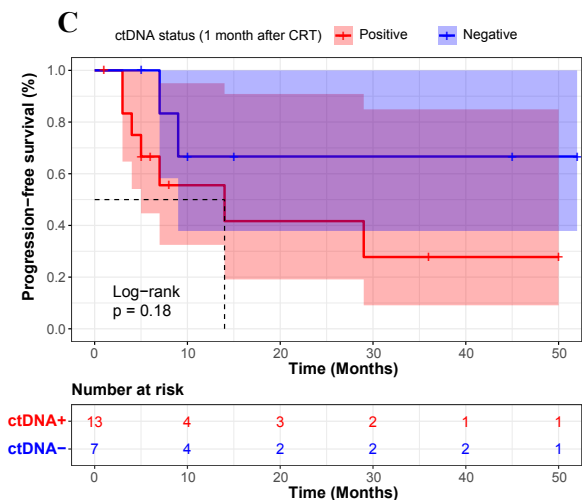

Supplement: Supplementary file 4 — Additional file 4: Supplementary Figure 4. The prognostic value of the TP2 time point. A-B Kaplan-Meier curve of progression-free survival (A) or overall survival (B) stratified by ctDNA detection status at TP2 in stage III patients of the discovery set. C Kaplan-Meier curve of progression-free survival stratified by ctDNA detection status at 1 month post-CRT in the external test cohort. A total of 20 independent stage III NSCLC patients whose plasma samples were collected at baseline, in the fourth week during CRT, and after 1 month of CRT were included in the analysis. [file 12943_2022_1590_MOESM4_ESM.pdf]

Supplementary Figure 5

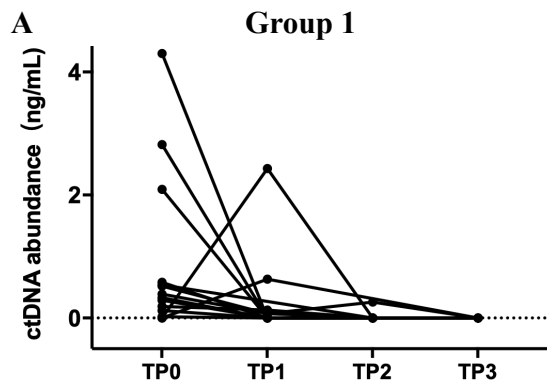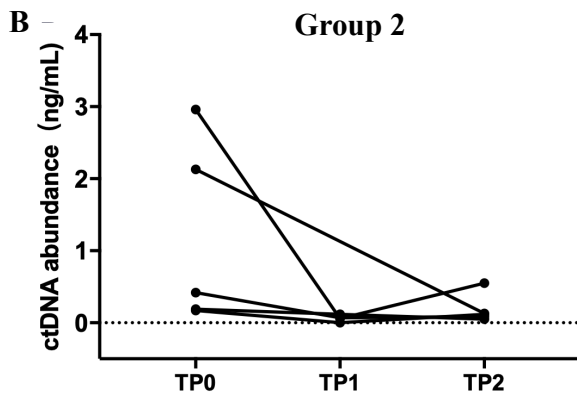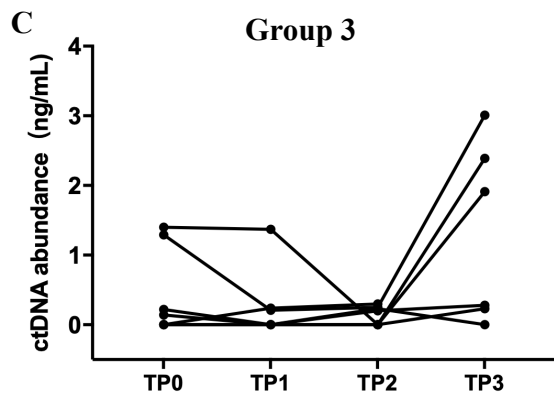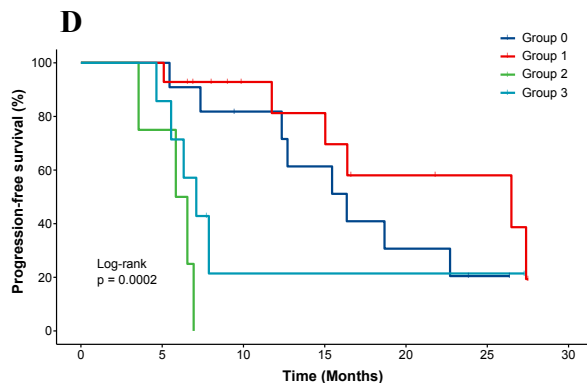

|         | Number at risk |   |   |   |   |   |   |
|---------|----------------|---|---|---|---|---|---|
| Group 0 | 11             | 8 | 6 | 3 | 1 | 0 | 0 |
| Group 1 | 14             | 7 | 1 | 4 | 3 | 0 | 0 |
| Group 2 | 4              | 3 | 0 | 0 | 0 | 0 | 0 |
| Group 3 | 7              | 6 | 1 | 1 | 1 | 1 | 0 |

Supplement: Supplementary file 5 — Additional file 5: Supplementary Figure 5. Patient grouping was based on the dynamic change in ctDNA in the discovery cohort. A Group 1 of patients whose ctDNA was detectable at TP0/TP1 and was cleared at the last follow-up. B Group 2 of patients whose ctDNA was decreased but still detectable at the last follow-up. C Group 3 of patients whose ctDNA was increased at the last follow-up compared with baseline. D Kaplan-Meier curve of progression-free survival stratified by ctDNA dynamic changes during disease surveillance in the discovery cohort. [file 12943_2022_1590_MOESM5_ESM.pdf]

# Supplementary Figure 6

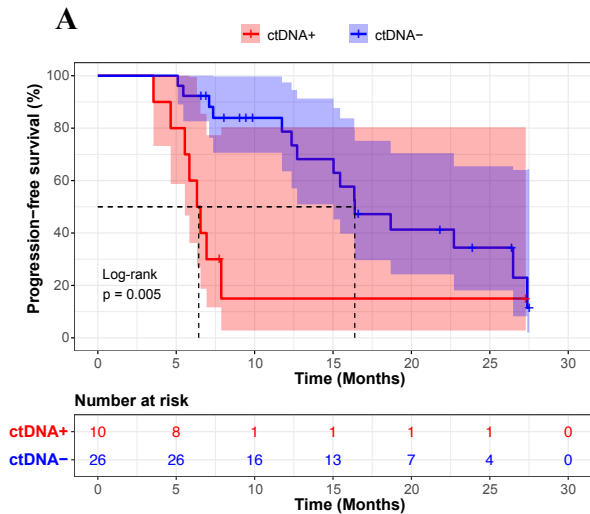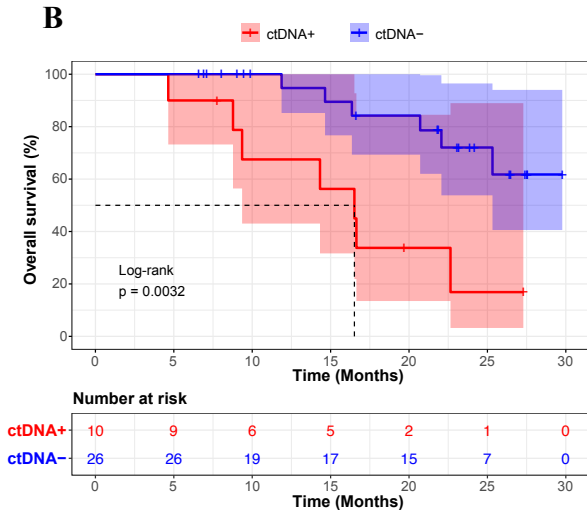

Supplement: Supplementary file 6 — Additional file 6: Supplementary Figure 6. The clearance of ctDNA at the last follow-up was associated with patients’ clinical outcomes. A-B Kaplan-Meier curve of progression-free survival (A) or overall survival (B) stratified by ctDNA detection status at the last follow-up before disease progression in the discovery set. [file 12943_2022_1590_MOESM6_ESM.pdf]
