# Supplementary material for: The clinical utility of dynamic ctDNA monitoring in inoperable localized NSCLC patients
Source: Mol Cancer. 2022 May 19;21:117. doi: 10.1186/s12943-022-01590-0 (PMC9118575; doi:10.1186/s12943-022-01590-0)
Supplement: Supplementary file 7 — Additional file 7: Supplementary methods. Supplementary Table 1. Demographic and clinical characteristics of the 55 NSCLC patients in the discovery set and 20 NSCLC patients in the test set. Supplementary Table 2. The relationship between baseline ctDNA detection and various clinical characteristics of the 55 NSCLC patients in the discovery set. Supplementary Table 3. The correlation between baseline ctDNA detection and clinical characteristics in 55 NSCLC patients of the discovery set. [file 12943_2022_1590_MOESM7_ESM.docx]

**Methods:**

**Patients and study design**

This study was approved by Institutional Review Boards of Cancer Hospital, Chinese Academy of Medical Sciences (ethical number: 19/098-1883), and all patients signed informed consent forms prior to sample collection. Fifty-nine patients who were diagnosed with NSCLC at Cancer Hospital, Chinese Academy of Medical Sciences from May 2018 to November 2020 were enrolled in this prospective study (clinical trial number: NCT04014465). The peripheral blood samples at various time points were collected and analyzed using targeted NGS of 474 cancer-related genes. Specifically, 55 out of 59 patients had baseline plasma samples collected at diagnosis (time point TP0) (**Fig. 1A**). Among the 55 patients, 8 patients received chemotherapy rather than chemoradiotherapy or radiotherapy (CRT/CT) due to liver/bone metastases or personal reasons, so these patients were only included in the baseline analyses but not the subsequent analyses. Forty-seven patients received CRT/RT as the front-line treatment. Most patients had their plasma samples collected during/post the treatment, including 39 patients at the fourth week of CRT/CT (time point TP1), 35 patients after 1 month of CRT/RT (time point TP2), 28 patients after 3 months of CRT/CT (time point TP3), and 13 patients at disease progression (PD) (time point TP4) (**Fig. 1A**). Besides ctDNA results, detailed pathological and clinical response data were also obtained for subsequent analysis. The median follow-up time was 26.4 months.

**DNA extraction and quantification**

The plasma fraction of peripheral blood samples was subjected to circulating free DNA (cfDNA) extraction with a Qiagen QIAamp Circulating Nucleic Acid Kit (Qiagen, Dusseldorf, Germany) [1]. Purified cfDNA samples were qualified using Nanodrop2000 (Thermo Fisher Scientific, Waltham, MA) and quantified using Qubit 2.0 using a dsDNA HS Assay Kit (Life Technologies, Waltham, MA).

**Library preparation and Hybridization capture**

Sequencing libraries were prepared using the KAPA Hyper Prep kit (KAPA Biosystems, Wilmington, MA) with an optimized manufacturer’s protocol [1]. Briefly, ∼50 ng of cfDNA was sequentially underwent end-repairing, A-tailing, and ligation with indexed adapters, followed by size selection using Agencourt AMPure XP beads (Beckman Coulter, Mississauga, Canada) and PCR amplification using KAPA Hyper DNA Library Prep Kit (KAPA Biosystems, Wilmington, MA). Target enrichment was performed using customized xGen lockdown probes (Integrated DNA Technologies) targeting 474 cancer- and radiotherapy-relevant genes (Radiotron®, Nanjing Geneseeq Technology Inc., Nanjing). The hybridization capture reaction was performed with Dynabeads M-279 (Life Technologies) and xGen Lockdown hybridization and wash kit (Integrated DNA Technologies) according to manufacturer’s protocols. Captured libraries were on-beads PCR amplified with Illumina p5 and p7 primers in KAPA HiFi HotStart ReadyMix (KAPA Biosystems), followed by purification using Agencourt AMPure XP beads. Libraries were quantified by qPCR using KAPA Library Quantification kit (KAPA Biosystems). Library fragment size was determined by Bioanalyzer 2100 (Agilent Technologies).

**Next-generation sequencing and data processing**

Sequencing was performed on the Illumina HiSeq4000 platform followed by data analysis as previously described [1 2]. In brief, sequencing data were analyzed by Trimmomatic [3] to remove low-quality (quality < 15) or N bases, and then mapped to the human reference genome hg19 using the Burrows-Wheeler Aligner (https://github.com/lh3/bwa/tree/master/bwakit). PCR duplicates were removed by Picard (available at: https://broadinstitute.github.io/picard/). The Genome Analysis Toolkit (GATK) (https://software.broadinstitute.org/gatk/) was used to perform local realignments around indels and base quality reassurance. Single nucleotide polymorphisms (SNPs) and indels were analyzed by VarScan2 [4] and Haplotype Caller/UniedGenotyper in GATK, with the mutant allele frequency (MAF) cutoff of 0.2% for cfDNA samples, and a minimum of three unique mutant reads. Common SNPs were excluded if they were present in > 1% population frequency in the 1000 Genomes Project or the Exome Aggregation Consortium (ExAC) 65,000 exomes database. The resulting mutation list was further filtered by an in-house list of recurrent artifacts based on a normal pool of whole blood samples. Gene fusions were identified by FACTERA [5]. The medium depth of coverage after the removal of PCR duplicates was >2,000X for plasma-derived cfDNA samples. ctDNA levels were calculated according to previous studies [6 7]: ctDNA abundance (ng/mL)=max ctDNA allele frequency*cfDNA concentration (ng/mL).

**Statistical analysis**

For survival data, Kaplan-Meier curves were analyzed using the log-rank test, and the statistical analyses were done in R (v.3.5.3). Comparisons were made using an unpaired two-tailed t-test, and analyses of variance (one-way ANOVA) was performed using Graphpad Prism V9.0. Two-sided *p* values of less than 0.05 were considered as statistically significant. Bonferroni method was used for multiple comparison correction.

**Reference:**

1. Yang Z, Yang N, Ou Q, et al. Investigating Novel Resistance Mechanisms to Third-Generation EGFR Tyrosine Kinase Inhibitor Osimertinib in Non-Small Cell Lung Cancer Patients. Clin Cancer Res 2018;**24**(13):3097-107 doi: 10.1158/1078-0432.CCR-17-2310[published Online First: Epub Date]|.

2. Shu Y, Wu X, Tong X, et al. Circulating Tumor DNA Mutation Profiling by Targeted Next Generation Sequencing Provides Guidance for Personalized Treatments in Multiple Cancer Types. Sci Rep 2017;**7**(1):583 doi: 10.1038/s41598-017-00520-1[published Online First: Epub Date]|.

3. Bolger AM, Lohse M, Usadel B. Trimmomatic: a flexible trimmer for Illumina sequence data. Bioinformatics 2014;**30**(15):2114-20 doi: 10.1093/bioinformatics/btu170[published Online First: Epub Date]|.

4. Koboldt DC, Zhang Q, Larson DE, et al. VarScan 2: somatic mutation and copy number alteration discovery in cancer by exome sequencing. Genome Res 2012;**22**(3):568-76 doi: 10.1101/gr.129684.111[published Online First: Epub Date]|.

5. Newman AM, Bratman SV, Stehr H, et al. FACTERA: a practical method for the discovery of genomic rearrangements at breakpoint resolution. Bioinformatics 2014;**30**(23):3390-3 doi: 10.1093/bioinformatics/btu549[published Online First: Epub Date]|.

6. Chaudhuri AA, Chabon JJ, Lovejoy AF, et al. Early Detection of Molecular Residual Disease in Localized Lung Cancer by Circulating Tumor DNA Profiling. Cancer Discov 2017;**7**(12):1394-403 doi: 10.1158/2159-8290.CD-17-0716[published Online First: Epub Date]|.

7. Mao X, Zhang Z, Zheng X, et al. Capture-Based Targeted Ultradeep Sequencing in Paired Tissue and Plasma Samples Demonstrates Differential Subclonal ctDNA-Releasing Capability in Advanced Lung Cancer. J Thorac Oncol 2017;**12**(4):663-72 doi: 10.1016/j.jtho.2016.11.2235[published Online First: Epub Date]|.

**Supplementary Table 1** Demographic and clinical characteristics of the 55 NSCLC patients in the discovery set and 20 NSCLC patients in the test set.

|  | **Number of patients (%)** | |
| --- | --- | --- |
|  | **Discovery set** | **Test set** |
| **Median age, years (range)** | 62 (40-82) | 62 (35-73) |
| **Gender** |  |  |
| Male | 45 (81.8) | 17 (85.0) |
| Female | 10 (18.2) | 3 (15.0) |
| **Pathology** |  |  |
| Adenocarcinoma | 25 (45.5) | 9 (45.0) |
| Squamous carcinoma | 28 (50.9) | 11 (55.0) |
| Others | 2 (3.6) | 0 (0.0) |
| **Stage** |  |  |
| I | 2 (3.6) | 0 (0.0) |
| II | 5 (9.1) | 0 (0.0) |
| IIIA | 12 (21.8) | 4 (20.0) |
| IIIB | 27 (49.1) | 10 (50.0) |
| IIIC | 9 (16.4) | 6 (30.0) |
| **T stage** |  |  |
| T1 | 10 (18.2) | 3 (15.0) |
| T2 | 13 (23.6) | 6 (30.0) |
| T3 | 15 (27.3) | 4 (20.0) |
| T4 | 17 (30.9) | 7 (35.0) |
| **N stage** |  |  |
| N0 | 6 (10.9) | 0 (0.0) |
| N1 | 8 (14.5) | 1 (5.0) |
| N2 | 19 (34.5) | 9 (45.0) |
| N3 | 22 (40) | 10 (50.0) |

**Supplementary Table 2** The relationship between baseline ctDNA detection and various clinical characteristics of the 55 NSCLC patients in the discovery set.

|  | **Total number of  patients (%)** | **Patients with positive  baseline ctDNA** | ***p* value** |
| --- | --- | --- | --- |
| **Total** | 55 | 34 |  |
| **Pathology** |  |  | 0.526 |
| Adenocarcinoma | 25 | 15 |  |
| Squamous carcinoma | 28 | 17 |  |
| Others | 2 | 2 |  |
| **Stage** |  |  | 0.0155 |
| I | 2 | 0 |  |
| II | 5 | 2 |  |
| IIIA | 12 | 4 |  |
| IIIB | 27 | 21 |  |
| IIIC | 9 | 7 |  |
| **T stage** |  |  | 0.0595 |
| T1 | 10 | 5 |  |
| T2 | 13 | 7 |  |
| T3 | 15 | 7 |  |
| T4 | 17 | 15 |  |
| **N stage** |  |  | 0.0113 |
| N0 | 6 | 2 |  |
| N1 | 8 | 3 |  |
| N2 | 19 | 11 |  |
| N3 | 22 | 18 |  |

**Supplementary table 3** The correlation between baseline ctDNA detection and clinical characteristics in 55 NSCLC patients of the discovery set.

| **Characteristics** | **cfDNA** | |  | **ctDNA** | |
| --- | --- | --- | --- | --- | --- |
|  | **cfDNA concentration (ng/ml)** | ***p* value** |  | **ctDNA abundance (ng/mL)** | ***p* value** |
| **Total** | 23.81±13.45 |  |  | 1.10±2.41 |  |
| **Pathology** |  |  |  |  |  |
| Adenocarcinoma | 24.22±17.41 | 0.583 |  | 0.76±1.02 | 0.564 |
| Squamous carcinoma | 23.97±8.98 |  |  | 1.32±3.18 |  |
| Others | 16.56±1.12 |  |  | 2.24±2.92 |  |
| **Stage** |  |  |  |  |  |
| I | 21.77±4.60 | 0.802 |  | 0.00±0.00 | 0.062 |
| II | 23.30±7.45 |  |  | 0.36±0.59 |  |
| IIIA | 24.45±9.96 |  |  | 0.18±0.37 |  |
| IIIB | 22.12±9.39 |  |  | 1.10±2.18 |  |
| IIIC | 28.79±25.58 |  |  | 3.06±4.2 |  |
| **T stage** |  |  |  |  |  |
| T1 | 19.23±6.24 | 0.501 |  | 0.39±0.88 | 0.029 |
| T2 | 25.15±9.83 |  |  | 0.46±0.6 |  |
| T3 | 22.53±8.65 |  |  | 0.31±0.72 |  |
| T4 | 26.62±19.99 |  |  | 2.71±3.8 |  |
| **N stage** |  |  |  |  |  |
| N0 | 23.40±7.11 | 0.482 |  | 0.08±0.16 | 0.421 |
| N1 | 19.38±6.71 |  |  | 0.43±0.62 |  |
| N2 | 27.51±9.66 |  |  | 1.17±2.57 |  |
| N3 | 22.35±18.08 |  |  | 1.61±2.94 |  |
